# Supplementary material for: End Sequence Analysis Toolkit (ESAT) expands the extractable information from single-cell RNA-seq data
Source: Genome Res. 2016 Oct;26(10):1397–410. doi: 10.1101/gr.207902.116 (PMC5052061; doi:10.1101/gr.207902.116)
Supplement: Supplemental Material [file supp_gr.207902.116_Supp_Materials.tar.gz › ESAT-esat_v0.1/doc/html/classbroad_1_1pda_1_1seq_1_1fastq_1_1_fastq_splitter_by_barcode-members.html]

ESAT: Member List


ESAT


- Main Page
- Related Pages
- Packages
- Classes
- Files

- Class List
- Class Index
- Class Hierarchy
- Class Members

All Classes Namespaces Files Functions Variables Enumerator Pages

- broad
- pda
- seq
- fastq
- FastqSplitterByBarcode

broad.pda.seq.fastq.FastqSplitterByBarcode Member List

This is the complete list of members for broad.pda.seq.fastq.FastqSplitterByBarcode, including all inherited members.

|  |  |  |
| --- | --- | --- |
| FastqSplitterByBarcode(String bcFile) | broad.pda.seq.fastq.FastqSplitterByBarcode |  |
| getPair1Parser() | broad.pda.seq.fastq.FastqSplitterByBarcode | protected |
| getPair2Parser() | broad.pda.seq.fastq.FastqSplitterByBarcode | protected |
| getRead1Trim3p() | broad.pda.seq.fastq.FastqSplitterByBarcode |  |
| getRead1Trim5p() | broad.pda.seq.fastq.FastqSplitterByBarcode |  |
| getRead2Trim3p() | broad.pda.seq.fastq.FastqSplitterByBarcode |  |
| getRead2Trim5p() | broad.pda.seq.fastq.FastqSplitterByBarcode |  |
| hasMore() | broad.pda.seq.fastq.FastqSplitterByBarcode |  |
| isAddToReadName() | broad.pda.seq.fastq.FastqSplitterByBarcode |  |
| isAtEnd() | broad.pda.seq.fastq.FastqSplitterByBarcode |  |
| isPaired() | broad.pda.seq.fastq.FastqSplitterByBarcode |  |
| main(String[] args) | broad.pda.seq.fastq.FastqSplitterByBarcode | static |
| setAddToReadName(boolean addToReadName) | broad.pda.seq.fastq.FastqSplitterByBarcode |  |
| setAtEnd(boolean atEnd) | broad.pda.seq.fastq.FastqSplitterByBarcode |  |
| setPair1Parser(FastqParser pair1Parser) | broad.pda.seq.fastq.FastqSplitterByBarcode | protected |
| setPair2Parser(FastqParser pair2Parser) | broad.pda.seq.fastq.FastqSplitterByBarcode | protected |
| setPaired(boolean paired) | broad.pda.seq.fastq.FastqSplitterByBarcode |  |
| setRead1Trim3p(int read1Trim3p) | broad.pda.seq.fastq.FastqSplitterByBarcode |  |
| setRead1Trim5p(int read1Trim5p) | broad.pda.seq.fastq.FastqSplitterByBarcode |  |
| setRead2Trim3p(int read2Trim3p) | broad.pda.seq.fastq.FastqSplitterByBarcode |  |
| setRead2Trim5p(int read2Trim5p) | broad.pda.seq.fastq.FastqSplitterByBarcode |  |
| start(String pair1Source, String outputDir, String pair2Source) | broad.pda.seq.fastq.FastqSplitterByBarcode |  |
| stop() | broad.pda.seq.fastq.FastqSplitterByBarcode |  |
| updateReadInfoWithBarcode(FastqSequence read, String bc) | broad.pda.seq.fastq.FastqSplitterByBarcode | protected |


---

Generated on Fri Aug 15 2014 15:11:02 for ESAT by  

 1.8.7
